# Ambulatory Anesthesia in an Adult Patient with Corrected Hypoplastic Left Heart Syndrome

**DOI:** 10.1155/2012/607140

**Published:** 2012-11-22

**Authors:** Jennifer Knautz, Yogen Asher, Mark C. Kendall, Robert Doty

**Affiliations:** Department of Anesthesiology, McGaw Medical Center, Feinberg School of Medicine, Northwestern University, Chicago, IL 60611, USA

## Abstract

With recent advancements in clinical science, an increasing number of patients with congenital heart defects are surviving into adulthood and presenting for noncardiac surgeries. We describe one such example of a 26-year-old patient with corrected hypoplastic left heart syndrome presenting for knee arthroscopy and performed under general anesthesia with preoperative ultrasound guided saphenous nerve block. In this case, we review the anesthetic implications of corrected single ventricle physiology, anesthetic implications, as well as discuss the technique and role of saphenous nerve block in patients undergoing knee arthroscopy.

## 1. Introduction

 Congenital heart defects occur in approximately 4–9/1000 live births. Advances in clinical science have resulted in an expected survival rate into adulthood of approximately 85% [1]. This allows adult patients with surgically corrected congenital heart defects to present for noncardiac surgery. A thorough understanding of their complex physiology is essential for safe anesthetic management. We present a case of a young adult patient with Fontan physiology who presented for ambulatory knee arthroscopy.

## 2. Case Report

A 26-year-old female with hypoplastic left heart syndrome (HLHS) presented to our ambulatory surgery center for knee arthroscopy for worsening knee pain. Her cardiac surgical history included undergoing a Norwood procedure at birth, Glen procedure at age 4 months, and Fontan procedure at age 5 years. Other medical history included hypertension, pacemaker insertion for sick sinus syndrome, lung arteriovenous malformations, and cirrhosis. Her exercise tolerance was greater than 4 metabolic equivalents, and her baseline oxygen saturation was 92–94% on room air. A summary of her preoperative evaluation can be seen in Table 1.

**Figure 1:**
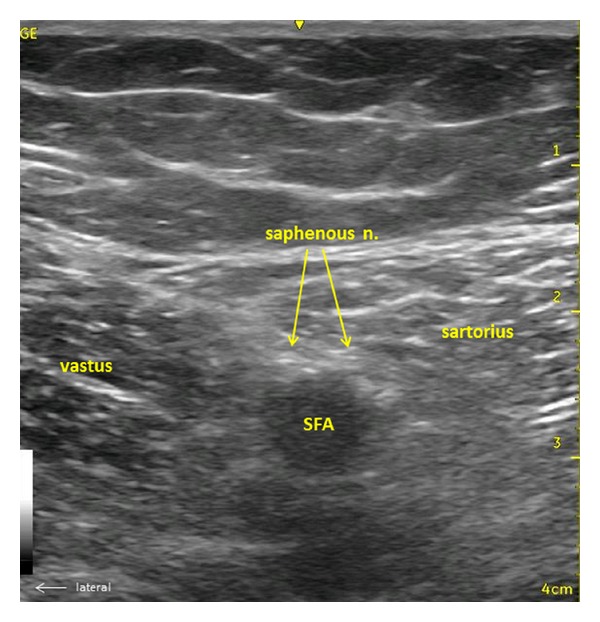
Sonoanatomy of saphenous nerve at mid-thigh.

Preoperative consultation with the surgical team indicated the desire to maintain motor function of the leg post op. The anesthetic plan was thus a general anesthetic with laryngeal mask airway (LMA) and a preoperative mid-thigh saphenous nerve block for postoperative analgesia. After peripheral intravenous (IV) access and standard monitors were placed, she received 250 mL of 5% albumin, 1 mg midazolam, and 2 liters oxygen via nasal cannula. After chlorhexidine prep, a mid-thigh, ultrasound-guided saphenous nerve block was performed with a 22 gauge 90 millimeter (mm) Pajunk block needle. Bupivacaine 0.5% 10 mL with epinephrine 1 : 300 k added was injected without complication (see Figure 1 for sonoanatomy of saphenous nerve). The patient was pre-oxygenated, and general anesthesia was induced with 50 mcg of fentanyl and propofol in IV increments of 50 mg (total 200 mg). An LMA was placed with immediate return of spontaneous ventilation; anesthesia was maintained with 1 MAC of sevoflurane, oxygen, and air. The patient remained hemodynamically stable during the case (blood pressure and heart rate were maintained within 20% of preoperative baseline values). An additional 250 mL of 5% albumin was infused during the case. At the end of the case the LMA was removed without complication. The patient was transferred to the PACU, and following anesthesia recovery, was discharged home after approximately 90 minutes without the need for additional opioids. The patient denied any nausea, had no episodes of emesis, and her pain was well controlled. Her hemodynamics remained stable postoperatively. Her pacemaker, interrogated before and after the procedure, remained functioning during the entire perioperative period.

## 3. Discussion

**Figure 2:**
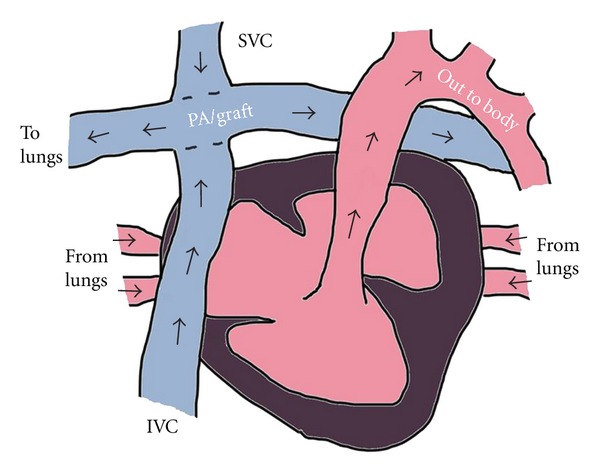
Simplified schematic depicting extracardiac Fontan physiology in HLHS. Deoxygenated blood is directly shunted to the lungs via graft to pulmonary arteries, then returned to heart via pulmonary veins where oxygenated blood can be pumped to the rest of the body.

Hypoplastic left heart syndrome is frequently repaired in 3 stages, with the final stage called a Fontan procedure where venous return is passively routed to the pulmonary arteries (see Figure 2). The cardiac output and pulmonary blood flow are determined by the transpulmonary gradient (the pressure difference between the systemic venous return and the pulmonary pressure). For this physiology to be effective, the goal systemic venous pressure should be approximately 10–15 mmHg with a pulmonary pressure of 5–10 mmHg, creating a driving pressure of 5–8 mmHg [2]. Because filling is passive, there must be adequate preload without obstruction to blood flow (patent grafts). Additionally, maintenance of sinus rhythm and a competent aortic valve are essential for adequate cardiac function.

Goals for our anesthetic management include maintenance of adequate preload, adequate oxygenation/ventilation to prevent pulmonary hypertension, adequate pain control, and avoiding nausea/vomiting, which could lead to dehydration and decreased preload postoperatively. While positive pressure has been used successfully in the past, its use may contribute to decreased venous return and increase pulmonary resistance, thus spontaneous ventilation should be used whenever possible. Approximately 4% of Fontan patients have a protein losing enteropathy that occurs via the GI tract. Thus, albumin was chosen for volume replacement in this case in order to maintain intravascular volume and to prevent third spacing of fluid given the diminished oncotic pressure from protein losing enteropathy. Hepatic dysfunction is common resulting from sluggish conduits and passive filling, and may necessitate evaluation prior to regional anesthesia.

The saphenous nerve is a distal branch of the posterior division of the femoral nerve, and branches into the infrapatellar branch (innervation to knee) and distal branches (antero-medial innervation of the lower leg/ankle). Mid-thigh, it lies between the vastus medialis and sartorius muscles adjacent to the superficial femoral artery (SFA). Saphenous nerve blocks have been used successfully to decrease pain scores and opioid consumption in patients undergoing knee arthroscopy [3] without interfering with quadricep function. In this case, the block was paramount in achieving intra- and postoperative pain control to avoid both the respiratory depressant and proemetic effects of opioids. In addition, given the possibility of liver dysfunction and increased risk of bleeding in this patient, this block is peripherally located. If bleeding was to occur from the block, compression could be used to control bleeding.

The combined effect of maintaining optimal cardiac function of this adult patient with HLHS under general anesthesia with diligent fluid management combined with preoperative placement of a saphenous nerve block to minimize the need for postoperative opioid use in the immediate per-operative period, provided a smooth uneventful course for an otherwise common outpatient surgical procedure.

## Figures and Tables

**Table 1:** Preoperative evaluation.

PMH	Hypoplastic left heart
	Sick sinus with pacemaker
	Hypertension
	Cirrhosis
	Lung arteriovenous malformations

PSH	Norwood (at birth)
	Glen (at 4 m)
	Fontan (at 5 y)
	Cholecystectomy
	Tonsillectomy
	Wrist surgery
	Esophagogastroduodenoscopy
	Cardiac cath

Meds	Spironolactone
	Digoxin
	Atenolol
	Aspirin
	Hydrochlorothiazide
	Losartan
	Pantoprazole
	Potassium chloride

Labs	Hemoglobin 17.5, platelets of 100 [nadir 50]
	AST 49, ALT 70
	INR of 1.3
	Na 123, K 3.7, Cr 0.73, HCO_3_ 23
	Albumin 4.8, total protein 7.2

EKG	Heart rate 79 bpm, atrial-paced with 1^o^ AV block, right access deviation,
	incomplete right bundle branch block, right ventricular hypertrophy,
	ST depression in precordial leads with T wave inversion (nonacute)

Echo	Grossly adequate myocardial function, no Fontan obstruction, unobstructed atrial septal defect, mild aortic regurgitation

Cardiac Cath	Patent superior vena cava to right pulmonary artery conduit with a saturation of 88%, mean pressure of 14 mmHg,
	inferior vena cava to left pulmonary artery conduit with a saturation of 85%, mean pressure of 14 mmHg

## References

[1] Bailey PD, Jobes DR (2009). The fontan patient. *Anesthesiology Clinics*.

[2] Khairy P, Poirier N, Mercier LA (2007). Univentricular heart. *Circulation*.

[3] Akkaya T, Ersan O, Ozkan D (2008). Saphenous nerve block is an effective regional technique for post-menisectomy pain. *Knee Surgery, Sports Traumatology, Arthroscopy*.

